# 4-[(*E*)-(3-Chloro-4-methyl­phen­yl)imino­meth­yl]-2-methoxy-3-nitro­phenyl acetate

**DOI:** 10.1107/S1600536811055590

**Published:** 2012-01-07

**Authors:** Deng-Cheng Su, Feng-Ting Wang, Cheng-Gong Mao, Shao-Song Qian

**Affiliations:** a School of Life Sciences, Shandong University of Technology, ZiBo 255049, People’s Republic of China

## Abstract

The title compound, C_17_H_15_ClN_2_O_5_, displays a *trans*-configuration with respect to the C=N double bond. The mol­ecule is twisted, the dihedral angle between the mean planes of the two benzene rings being 18.70 (12)°. The nitro, meth­oxy and acetyl groups are oriented at 80.70 (11), 35.2 (2) and 72.35 (10)°, respectively, to the benzene ring to which they are bonded. The crystal structure is stabilized by weak C—H⋯O hydrogen-bonding contacts.

## Related literature

For background to Schiff bases in coordination chemistry, see: Bhatia *et al.* (1981 ▶); Costamagna *et al.* (1992 ▶). For a related structure, see: Qian & Liu (2010 ▶).
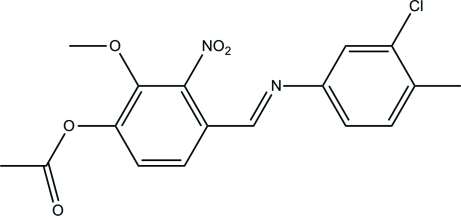


## Experimental

### 

#### Crystal data

C_17_H_15_ClN_2_O_5_
*M* *_r_* = 362.76
Triclinic, 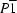

*a* = 7.035 (6) Å
*b* = 7.672 (6) Å
*c* = 17.272 (14) Å
α = 83.477 (8)°
β = 84.994 (8)°
γ = 66.697 (7)°
*V* = 849.7 (12) Å^3^
*Z* = 2
Mo *K*α radiation
μ = 0.26 mm^−1^
*T* = 296 K
0.26 × 0.23 × 0.21 mm

#### Data collection

Bruker APEXII CCD diffractometer
Absorption correction: multi-scan (*SADABS*; Bruker, 2004 ▶) *T*_min_ = 0.936, *T*_max_ = 0.948
5579 measured reflections
3083 independent reflections
2207 reflections with *I* > 2σ(*I*)
*R*_int_ = 0.022

#### Refinement

*R*[*F* ^2^ > 2σ(*F* ^2^)] = 0.045
*wR*(*F* ^2^) = 0.122
*S* = 1.05
3083 reflections
229 parameters
H-atom parameters constrained
Δρ_max_ = 0.17 e Å^−3^
Δρ_min_ = −0.23 e Å^−3^

### 

Data collection: *APEX2* (Bruker, 2004 ▶); cell refinement: *SAINT* (Bruker, 2004 ▶); data reduction: *SAINT*; program(s) used to solve structure: *SHELXS97* (Sheldrick, 2008 ▶); program(s) used to refine structure: *SHELXL97* (Sheldrick, 2008 ▶); molecular graphics: *SHELXTL* (Sheldrick, 2008 ▶); software used to prepare material for publication: *SHELXTL*.

## Supplementary Material

Crystal structure: contains datablock(s) global, I. DOI: 10.1107/S1600536811055590/pv2498sup1.cif

Structure factors: contains datablock(s) I. DOI: 10.1107/S1600536811055590/pv2498Isup2.hkl

Supplementary material file. DOI: 10.1107/S1600536811055590/pv2498Isup3.cml

Additional supplementary materials:  crystallographic information; 3D view; checkCIF report

## Figures and Tables

**Table 1:** Hydrogen-bond geometry (Å, °)

*D*—H⋯*A*	*D*—H	H⋯*A*	*D*⋯*A*	*D*—H⋯*A*
C12—H12⋯O1^i^	0.93	2.58	3.431 (3)	153

Symmetry code: (i) 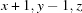.

**Fig. 1.**
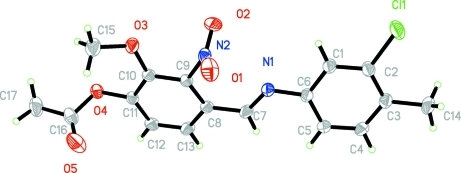
The structure of the title molecule showing the atom-labelling scheme. Displacement ellipsoids are drawn at the 30% probability level.

**Fig. 2.**
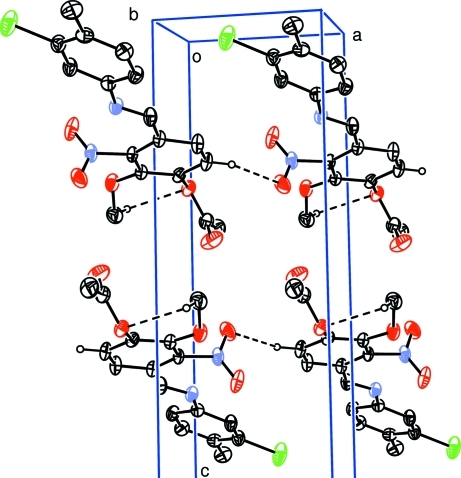
The packing diagram for the title compound; hydrogen-bonding interactions are represented by dashed lines.

## supplementary crystallographic information

### Comment

Schiff bases play an important role in the development of coordination chemistry related to catalysis and enzymatic reactions, magnetism and molecular architectures (Costamagna *et al.*, 1992; Bhatia *et al.*, 1981). As an extension of work on the structural characterization of Schiff base compounds, we report the synthesis and crystal structure of the title compound in this article.

The title compound (Fig. 1) assumes an E conformation about the C═N double bond. The molecule is twisted, with the dihedral angle between the two benzene rings being 18.70 (12)°. The nitro (N2/O1/O2), methoxy (O3/C15) and ethanone (O4/O5/C16/C17) groups are oriented with respect to the benzene ring (C8–C13) to which they are bonded, at 80.70 (11), 35.2 (2) and 72.35 (10) °, respectively. The crystal structure is stabilized by π-π interactions [centroid-centroid distance = 3.886 (4) Å ] and weak C12—H12···O1 hydrogen bonding contacts.

The bond lengths and bond angles in the title compound are comparable to the corresponding bond lengths and bond angles observed in a closely related compound (Qian & Liu, 2010).

### Experimental

4-Formyl-2-methoxy-3-nitrophenyl acetate (0.0995 g) and 3-chloro-4-methylaniline (0.0706 g) were dissolved in methanol (20 mL). The mixture was stirred at room temperature for 45 mins to give a clear solution. The solution was allowed to stand in the air for 3 days. Yellow block-shaped single crystals of the title compound suitable for X-ray diffraction analysis were obtained at the bottom of the vessel.

### Refinement

All H atoms were placed in geometrical positions and constrained to ride on their parent atoms with C—H distances in the range 0.93–0.96 Å, in a riding mode, with *U*_iso_(H) = k*U*_eq_(C), where k = 1.5 for methyl and 1.2 for all other H atoms.

### Crystal data

**Table:**

C_17_H_15_ClN_2_O_5_	*Z* = 2
*M**_r_* = 362.76	*F*(000) = 376
Triclinic, *P*1	*D*_x_ = 1.418 Mg m^−^^3^
Hall symbol: -P 1	Mo *K*α radiation, λ = 0.71073 Å
*a* = 7.035 (6) Å	Cell parameters from 3083 reflections
*b* = 7.672 (6) Å	θ = 2.4–25.5°
*c* = 17.272 (14) Å	µ = 0.26 mm^−^^1^
α = 83.477 (8)°	*T* = 296 K
β = 84.994 (8)°	Block, yellow
γ = 66.697 (7)°	0.26 × 0.23 × 0.21 mm
*V* = 849.7 (12) Å^3^	

### Data collection

**Table:**

Bruker APEXII CCD diffractometer	3083 independent reflections
Radiation source: fine-focus sealed tube	2207 reflections with *I* > 2σ(*I*)
graphite	*R*_int_ = 0.022
φ and ω scans	θ_max_ = 25.5°, θ_min_ = 2.4°
Absorption correction: multi-scan (*SADABS*; Bruker, 2004)	*h* = −8→8
*T*_min_ = 0.936, *T*_max_ = 0.948	*k* = −9→9
5579 measured reflections	*l* = −20→17

### Refinement

**Table:**

Refinement on *F*^2^	Primary atom site location: structure-invariant direct methods
Least-squares matrix: full	Secondary atom site location: difference Fourier map
*R*[*F*^2^ > 2σ(*F*^2^)] = 0.045	Hydrogen site location: inferred from neighbouring sites
*wR*(*F*^2^) = 0.122	H-atom parameters constrained
*S* = 1.05	*w* = 1/[σ^2^(*F*_o_^2^) + (0.0433*P*)^2^ + 0.4254*P*] where *P* = (*F*_o_^2^ + 2*F*_c_^2^)/3
3083 reflections	(Δ/σ)_max_ < 0.001
229 parameters	Δρ_max_ = 0.17 e Å^−^^3^
0 restraints	Δρ_min_ = −0.23 e Å^−^^3^

### Special details

**Table:**

Geometry. All esds (except the esd in the dihedral angle between two l.s. planes) are estimated using the full covariance matrix. The cell esds are taken into account individually in the estimation of esds in distances, angles and torsion angles; correlations between esds in cell parameters are only used when they are defined by crystal symmetry. An approximate (isotropic) treatment of cell esds is used for estimating esds involving l.s. planes.
Refinement. Refinement of F^2^ against ALL reflections. The weighted R-factor wR and goodness of fit S are based on F^2^, conventional R-factors R are based on F, with F set to zero for negative F^2^. The threshold expression of F^2^ > 2sigma(F^2^) is used only for calculating R-factors(gt) etc. and is not relevant to the choice of reflections for refinement. R-factors based on F^2^ are statistically about twice as large as those based on F, and R- factors based on ALL data will be even larger.

### Fractional atomic coordinates and isotropic or equivalent isotropic displacement parameters (Å^2^)

**Table:**

	*x*	*y*	*z*	*U*_iso_*/*U*_eq_	
Cl1	−0.50222 (11)	1.23475 (11)	0.05852 (6)	0.0880 (3)	
O1	−0.3219 (3)	0.5157 (3)	0.34263 (13)	0.0728 (6)	
O2	−0.3928 (3)	0.4212 (3)	0.23925 (14)	0.0733 (6)	
O3	−0.2466 (2)	0.0650 (2)	0.34480 (11)	0.0563 (5)	
O4	0.1492 (2)	−0.2520 (2)	0.35298 (10)	0.0472 (4)	
O5	0.2885 (4)	−0.2451 (3)	0.46343 (13)	0.0891 (8)	
N1	−0.0690 (3)	0.6082 (3)	0.20244 (12)	0.0450 (5)	
N2	−0.2784 (3)	0.4109 (3)	0.28994 (14)	0.0490 (5)	
C1	−0.2521 (3)	0.9075 (3)	0.13350 (15)	0.0491 (6)	
H1	−0.3684	0.8771	0.1395	0.059*	
C2	−0.2654 (4)	1.0797 (3)	0.09522 (15)	0.0491 (6)	
C3	−0.0985 (4)	1.1342 (3)	0.08453 (14)	0.0459 (6)	
C4	0.0867 (4)	1.0021 (4)	0.11302 (15)	0.0510 (6)	
H4	0.2035	1.0317	0.1061	0.061*	
C5	0.1053 (4)	0.8288 (3)	0.15121 (15)	0.0472 (6)	
H5	0.2328	0.7443	0.1692	0.057*	
C6	−0.0665 (3)	0.7799 (3)	0.16295 (13)	0.0405 (5)	
C7	0.0954 (3)	0.4730 (3)	0.22155 (15)	0.0469 (6)	
H7	0.2197	0.4885	0.2100	0.056*	
C8	0.1029 (3)	0.2917 (3)	0.26103 (14)	0.0411 (5)	
C9	−0.0705 (3)	0.2553 (3)	0.29035 (13)	0.0377 (5)	
C10	−0.0619 (3)	0.0795 (3)	0.32370 (13)	0.0390 (5)	
C11	0.1336 (3)	−0.0677 (3)	0.32809 (14)	0.0404 (5)	
C12	0.3094 (3)	−0.0359 (3)	0.30170 (15)	0.0492 (6)	
H12	0.4389	−0.1349	0.3063	0.059*	
C13	0.2947 (3)	0.1401 (3)	0.26888 (16)	0.0505 (6)	
H13	0.4148	0.1587	0.2515	0.061*	
C14	−0.1131 (5)	1.3253 (4)	0.04590 (17)	0.0626 (8)	
H14A	0.0227	1.3280	0.0397	0.094*	
H14B	−0.2006	1.4239	0.0778	0.094*	
H14C	−0.1706	1.3456	−0.0043	0.094*	
C15	−0.2664 (4)	−0.0543 (4)	0.41121 (17)	0.0644 (8)	
H15A	−0.2223	−0.1825	0.3972	0.097*	
H15B	−0.4086	−0.0101	0.4301	0.097*	
H15C	−0.1817	−0.0511	0.4513	0.097*	
C16	0.2390 (4)	−0.3320 (4)	0.42227 (17)	0.0568 (7)	
C17	0.2612 (5)	−0.5342 (4)	0.43672 (19)	0.0748 (9)	
H17A	0.2924	−0.5781	0.4902	0.112*	
H17B	0.3714	−0.6114	0.4033	0.112*	
H17C	0.1340	−0.5434	0.4260	0.112*	

### Atomic displacement parameters (Å^2^)

**Table:**

	*U*^11^	*U*^22^	*U*^33^	*U*^12^	*U*^13^	*U*^23^
Cl1	0.0514 (4)	0.0650 (5)	0.1311 (8)	−0.0151 (3)	−0.0205 (4)	0.0421 (5)
O1	0.0657 (13)	0.0466 (11)	0.0789 (15)	0.0036 (9)	0.0188 (11)	−0.0090 (11)
O2	0.0380 (10)	0.0623 (12)	0.1146 (18)	−0.0145 (9)	−0.0277 (11)	0.0122 (11)
O3	0.0386 (9)	0.0504 (10)	0.0747 (13)	−0.0175 (8)	−0.0028 (8)	0.0165 (9)
O4	0.0476 (9)	0.0301 (8)	0.0579 (11)	−0.0087 (7)	−0.0108 (8)	0.0025 (7)
O5	0.121 (2)	0.0643 (14)	0.0713 (15)	−0.0178 (13)	−0.0387 (14)	−0.0035 (11)
N1	0.0378 (10)	0.0385 (11)	0.0580 (13)	−0.0158 (8)	−0.0069 (9)	0.0048 (9)
N2	0.0326 (10)	0.0328 (11)	0.0714 (15)	−0.0065 (8)	0.0028 (11)	0.0088 (11)
C1	0.0351 (12)	0.0438 (14)	0.0662 (17)	−0.0165 (10)	−0.0007 (11)	0.0069 (12)
C2	0.0408 (13)	0.0429 (14)	0.0576 (16)	−0.0130 (10)	−0.0023 (11)	0.0067 (12)
C3	0.0551 (14)	0.0434 (13)	0.0429 (14)	−0.0247 (11)	−0.0005 (11)	0.0003 (11)
C4	0.0502 (14)	0.0562 (15)	0.0571 (16)	−0.0331 (12)	−0.0053 (12)	0.0024 (12)
C5	0.0400 (12)	0.0464 (14)	0.0578 (15)	−0.0200 (11)	−0.0085 (11)	0.0024 (12)
C6	0.0407 (12)	0.0370 (12)	0.0444 (13)	−0.0168 (10)	−0.0012 (10)	−0.0007 (10)
C7	0.0319 (12)	0.0392 (13)	0.0702 (17)	−0.0153 (10)	−0.0014 (11)	−0.0024 (12)
C8	0.0311 (11)	0.0332 (12)	0.0575 (15)	−0.0103 (9)	−0.0066 (10)	−0.0031 (10)
C9	0.0276 (10)	0.0284 (11)	0.0510 (14)	−0.0039 (8)	−0.0048 (10)	−0.0025 (10)
C10	0.0319 (11)	0.0354 (12)	0.0474 (14)	−0.0105 (9)	−0.0040 (10)	−0.0023 (10)
C11	0.0423 (12)	0.0276 (11)	0.0478 (14)	−0.0087 (9)	−0.0110 (10)	−0.0014 (10)
C12	0.0313 (12)	0.0365 (13)	0.0714 (17)	−0.0030 (10)	−0.0127 (11)	−0.0022 (12)
C13	0.0290 (11)	0.0395 (13)	0.0805 (19)	−0.0104 (10)	−0.0060 (11)	−0.0035 (12)
C14	0.0789 (19)	0.0525 (16)	0.0637 (18)	−0.0371 (15)	−0.0055 (15)	0.0103 (14)
C15	0.0564 (16)	0.0651 (18)	0.0666 (19)	−0.0235 (14)	0.0067 (14)	0.0065 (15)
C16	0.0533 (15)	0.0428 (15)	0.0562 (17)	−0.0006 (12)	−0.0052 (13)	0.0015 (13)
C17	0.0739 (19)	0.0481 (16)	0.084 (2)	−0.0107 (14)	−0.0018 (16)	0.0189 (15)

### Geometric parameters (Å, °)

**Table:**

Cl1—C2	1.743 (3)	C7—C8	1.461 (3)
O1—N2	1.221 (3)	C7—H7	0.9300
O2—N2	1.215 (3)	C8—C13	1.395 (3)
O3—C10	1.362 (3)	C8—C9	1.397 (3)
O3—C15	1.417 (3)	C9—C10	1.386 (3)
O4—C16	1.366 (3)	C10—C11	1.394 (3)
O4—C11	1.394 (3)	C11—C12	1.382 (3)
O5—C16	1.186 (3)	C12—C13	1.372 (3)
N1—C7	1.250 (3)	C12—H12	0.9300
N1—C6	1.419 (3)	C13—H13	0.9300
N2—C9	1.478 (3)	C14—H14A	0.9600
C1—C2	1.381 (3)	C14—H14B	0.9600
C1—C6	1.383 (3)	C14—H14C	0.9600
C1—H1	0.9300	C15—H15A	0.9600
C2—C3	1.386 (4)	C15—H15B	0.9600
C3—C4	1.386 (3)	C15—H15C	0.9600
C3—C14	1.508 (3)	C16—C17	1.490 (4)
C4—C5	1.378 (3)	C17—H17A	0.9600
C4—H4	0.9300	C17—H17B	0.9600
C5—C6	1.393 (3)	C17—H17C	0.9600
C5—H5	0.9300		
			
C10—O3—C15	120.89 (19)	O3—C10—C9	116.62 (18)
C16—O4—C11	117.65 (19)	O3—C10—C11	126.4 (2)
C7—N1—C6	121.1 (2)	C9—C10—C11	116.9 (2)
O2—N2—O1	125.6 (2)	C12—C11—C10	120.7 (2)
O2—N2—C9	118.1 (2)	C12—C11—O4	119.86 (19)
O1—N2—C9	116.3 (2)	C10—C11—O4	119.2 (2)
C2—C1—C6	120.3 (2)	C13—C12—C11	120.7 (2)
C2—C1—H1	119.9	C13—C12—H12	119.6
C6—C1—H1	119.9	C11—C12—H12	119.6
C1—C2—C3	122.8 (2)	C12—C13—C8	121.2 (2)
C1—C2—Cl1	118.53 (19)	C12—C13—H13	119.4
C3—C2—Cl1	118.66 (19)	C8—C13—H13	119.4
C4—C3—C2	115.7 (2)	C3—C14—H14A	109.5
C4—C3—C14	120.9 (2)	C3—C14—H14B	109.5
C2—C3—C14	123.4 (2)	H14A—C14—H14B	109.5
C5—C4—C3	122.8 (2)	C3—C14—H14C	109.5
C5—C4—H4	118.6	H14A—C14—H14C	109.5
C3—C4—H4	118.6	H14B—C14—H14C	109.5
C4—C5—C6	120.2 (2)	O3—C15—H15A	109.5
C4—C5—H5	119.9	O3—C15—H15B	109.5
C6—C5—H5	119.9	H15A—C15—H15B	109.5
C1—C6—C5	118.1 (2)	O3—C15—H15C	109.5
C1—C6—N1	116.1 (2)	H15A—C15—H15C	109.5
C5—C6—N1	125.7 (2)	H15B—C15—H15C	109.5
N1—C7—C8	123.6 (2)	O5—C16—O4	122.1 (3)
N1—C7—H7	118.2	O5—C16—C17	127.0 (3)
C8—C7—H7	118.2	O4—C16—C17	110.9 (3)
C13—C8—C9	116.4 (2)	C16—C17—H17A	109.5
C13—C8—C7	118.9 (2)	C16—C17—H17B	109.5
C9—C8—C7	124.71 (19)	H17A—C17—H17B	109.5
C10—C9—C8	124.07 (18)	C16—C17—H17C	109.5
C10—C9—N2	115.70 (19)	H17A—C17—H17C	109.5
C8—C9—N2	120.18 (19)	H17B—C17—H17C	109.5

### Hydrogen-bond geometry (Å, °)

**Table:**

*D*—H···*A*	*D*—H	H···*A*	*D*···*A*	*D*—H···*A*
C15—H15A···O4	0.96	2.52	2.861 (4)	101
C12—H12···O1^i^	0.93	2.58	3.431 (3)	153

Symmetry codes: (i) *x*+1, *y*−1, *z*.
